# Laparoscopic hemi-hysterectomy in a noncommunicating uterine horn: The critical steps to be considered

**DOI:** 10.4274/tjod.galenos.2020.01709

**Published:** 2020-07-29

**Authors:** Şadıman Kıykaç Altınbaş, Ömer Lütfi Tapısız, Mehmet Ünsal, Özlem Moraloğlu Tekin

**Affiliations:** 1 University of Health Sciences Turkey, Etlik Zübeyde Hanım Women’s Health Training and Research Hospital, Ankara, Turkey

**Keywords:** Uterine horn, laparoscopy, hemi-hysterectomy

## Abstract

Various congenital anomalies of the female tract such as agenesis, vertical or lateral fusion failure, and canalization failure occur when the normal development of the Müllerian duct disrupts in any stage of developmental milestones. A cavitated non-communicating rudimentary horn is reported in about 20%-25% of women with unicornuate uterus. A 36-year-old patient, gravida 2 para 2, was admitted to the hospital with a complaint of worsening lower abdominal pain occurring on each menses for 8 months. A 6-cm accessory cavitated left uterine mass suggestive of hematometra was shown on ultrasound examination. It was decided to perform hemi-hysterectomy to remove the left uterine horn by the laparoscopic route. Here we aimed to demonstrate the laparoscopic management of a rudimentary horn case and emphasize the crucial steps that surgeons should safely perform during the operation.

## Introduction

Various congenital anomalies of the female tract such as agenesis, vertical or lateral fusion failure, and canalization failure occur when the normal development of the Müllerian duct disrupts any stage of the developmental milestones. The unicornuate uterus is caused by the normal maturation of only one Müllerian duct. In some cases, the contralateral Müllerian duct is absent or partially develops, called a rudimentary horn, which may or may not communicate with the normally developed one Müllerian duct, called a unicornuate uterus.

A cavitated non-communicating rudimentary horn is reported in about 20%-25% of women with a unicornuate uterus^(1,2)^. The symptoms differ with the functionality of the endometrial cavity, and the patients’ symptoms depend on the presence of an obstructive anomaly causing pain regarding hematometra, hematosalpinx, or endometriosis due to retrograde menstruation^(3)^. There have been studies reporting reproductive outcome improvement by removing the rudimentary horn, but information on such approach is still lacking.

In the present case, we aimed to discuss the laparoscopic management of a multiparous patient with a rudimentary horn presenting with dysmenorrhea and dyspareunia symptoms in her late 30s.

## Case Report

**Figure 1:**
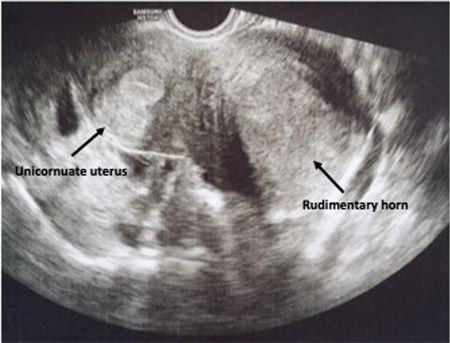
Cavitated left uterine mass suggestive of hematometra

**Figure 2:**
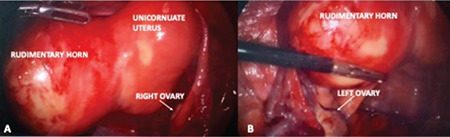
**A)** The abdominal cavity revealing a left noncommunicating rudimentary horn tightly resided on the lateral abdominal wall and **B)** two normal-looking ovaries and tubes

A 36-year-old patient, gravida 2 para 2, was admitted to the hospital with a complaint of worsening lower abdominal pain occurring on each menses for 8 months. Her external and internal genitalia, including the cervix, were normal except for the 6 cm accessory cavitated left uterine mass suggestive of hematometra that is compressing the urinary bladder without any other genitourinary system pathologies shown on ultrasound examination (Figure 1). Diagnostic hysteroscopy revealed a single cervix without any vaginal malformations and a relatively small uterine cavity with right tubal ostium and without left tubal ostium. It was decided to remove the left uterine horn (Class U4a/Hemi Uterus)^(4)^ by the laparoscopic route. Evaluation of the abdominal cavity revealed a left non-communicating rudimentary horn tightly residing on the lateral abdominal wall and two grossly normal ovaries and tubes (Figure 2). A probable occult occlusion of the tube might be present, and this tubal occlusion might cause this late occurrence. However, neither endometriosis nor any prior tubal or abdominal operation history was noted.

**Figure 3:**
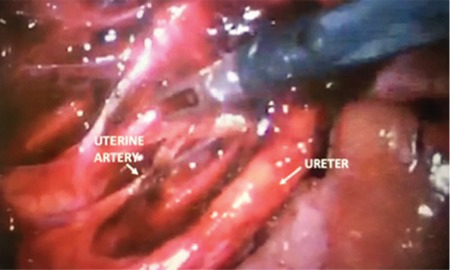
Dissection of the retroperitoneal space showing the ligated left uterine artery and the left ureter

First, to remove the fallopian tubes from the left uterine horn, they were coagulated and divided by careful tissue transection. Second, the vesicouterine peritoneum was divided to create the bladder flap from the cervix and the left uterine horn. Third, dissection of the retroperitoneal space beneath the round ligament to identify the ureter and the left hypogastric artery branches was performed. The broad ligament was fenestrated to lateralize the left ureter and facilitate transection of the utero-ovarian pedicle. The retroperitoneum was dissected, and the ureter tract was followed. Posterior peritoneum was also opened to create distance from the ureter and provide a place for the division of the horn by a monopolar hook. After the dissection and coagulation of the left uterine artery at the origin of the left hypogastric artery to minimize the bleeding during excision of the uterine horn by an advanced bipolar energy device (Figure 3), the resection of the rudimentary horn was achieved using a monopolar hook^(5)^. After controlling the bleeding and irrigating and suctioning the abdominal cavity, no other hemostasis sutures were required, and the operation was completed successfully. The patient was discharged on the first postoperative day, and normal regular menstrual cycles without any pain and complaints during the 6 and 12 months after the surgery were noted.

The patient signed an informed consent that allowed us to use her data.

## Discussion

Since the first documentation of laparoscopic removal of the rudimentary horn in 1990 by Canis et al.^(6)^, laparoscopy has become the standard treatment with proven advantages, including short operative time and hospital admission duration and less blood loss and postoperative pain. Although laparoscopic excision of the rudimentary uterine horn seems to be an effective and feasible surgical approach in experienced hands, it should always be remembered that anatomical landmarks and retroperitoneal space must be defined as the cleavage planes of the uterine horn and that the unicornuate uterus is not well defined all the time.

Two anatomical variations in the attachment of the rudimentary horn to the unicornuate uterus were reported, and one can be attached by either a band of tissue or firmly to the latter. When no fusion occurs with the contralateral duct, a fibrous or fibromuscular band connects the two horns^(7)^. Here, the rudimentary horn was attached firmly to the right unicornuate uterus and the left sidewall. When the borders are firm and not easy to distinguish, it can be difficult to remove the horn. To minimize the risk of penetration into the cavity of the hemiuterus while laparoscopic dissection, a hysteroscopic transillumination technique was performed in three cases by Nezhat et al.^(8)^. In a recent study by Jan et al.^(9)^, this technique was detailed in a technical video demonstrating the hysteroscopic transillumination of the plane of the dissection between the rudimentary horn and the uterus. At the beginning of the operation, we first performed a hysteroscopic evaluation of the uterine cavity to visualize the relation.

Besides, the most important point about these anomalies is the preoperative evaluation of the patients because other probable concomitant female reproductive tract anomalies and renal and skeletal system abnormalities may co-occur with Müllerian anomalies^(10)^. Anomaly observation of these coexisting defects should be performed, and the right treatment should be planned after defining the anatomy of pathology as possible with proper imaging techniques such as an ultrasound scan and magnetic resonance imaging. Regarding the definition of the anatomy of the pathology, type of attachment, and communication between the rudimentary horn and the hemiuterus considering other probable pathology exclusions like myomas or an obstructed hemivagina, the right treatment option should be discussed and performed.

After defining the anatomy of pathology, type of attachment, and communication between the rudimentary horn and the hemiuterus, the right treatment option should be discussed and performed. Here, preoperative examinations including transvaginal ultrasonography and an intravenous pyelogram showing the normal kidneys and ureters were performed.

Another step of the operation depends on the blood supply of the rudimentary horn as it may not always be from the ipsilateral uterine artery but also from the contralateral uterine artery^(2)^. Therefore, the ligation of the major blood supply from the uterine artery at the isthmus level may be impossible to achieve, so the dissection of the retroperitoneal space to develop a plane for the ipsilateral ureter lying adjacent to the vascular supply of the uterine horn is of great importance to prevent injury of the uterine artery during coagulation and ligation at the level of the hypogastric origin. A monopolar hook may not be sufficient while resecting the rudimentary horn; thus, advanced bipolar energy devices are needed to control the bleeding as the rudimentary horn may receive blood from the myometrial arcuate arteries of the contralateral uterine artery^(2)^. In cases where a firmly attached horn is present, the laparoscopic surgeon should handle large myometrial defect with sutures for reconstruction after removal of the horn^(9)^. Moreover, if the patient has a desire for a future pregnancy, the myometrial defect should also be sutured to avoid probable uterine rupture. The removal of the ipsilateral fallopian tube should always be performed to prevent a tubal pregnancy and cancer development.

In conclusion, a careful preoperative examination should be performed to detect the anatomical subtype, attachment type, and other coexisting genital malformations. Although the laparoscopic approach in these abnormalities seems to be effective and feasible, it should always be remembered that the anatomical landmarks and retroperitoneal space must be defined, and careful hemostasis must be performed in every step of the operation.
